# A Longitudinal Examination of the Association Between Meaning in Life, Resilience, and Mental Well-Being in Times of Coronavirus Pandemic

**DOI:** 10.3389/fpsyg.2021.645597

**Published:** 2021-04-28

**Authors:** Gökmen Arslan, Murat Yıldırım

**Affiliations:** ^1^Department of Psychological Counseling and Guidance, Mehmet Akif Ersoy University, Burdur, Turkey; ^2^International Network on Personal Meaning, Toronto, ON, Canada; ^3^Department of Psychology, Ağrı İbrahim Çeçen University, Agri, Turkey; ^4^Department of Neuroscience, Psychology and Behaviour, University of Leicester, Leicester, United Kingdom

**Keywords:** coronavirus pandemic, meaning in life, resilience, mental well-being, existential positive psychology

## Abstract

The coronavirus disease possesses an important threat to people's health and well-being. The purpose of the present study is to longitudinally examine whether meaning in life before the pandemic increases resilience and mental well-being during the coronavirus pandemic. The sample of the study comprised 172 young adults (72% women) in a public university in an urban city of Turkey. Participants ranged in age between 18 and 40 years (*M* = 20.87, *SD* = 3.92). Mediation analyses were performed to examine the impacts of meaning in life on the outcomes across the two waves of data. Findings from these analyses revealed that meaning in life before the pandemic had a significant predictive effect on resilience and mental well-being of young adults during the coronavirus disease. Resilience also mediated the impacts of meaning in life on mental well-being indicators at the second time. These results suggest the importance of meaning-based preventions and interventions designed to build up resilience experiences for improving psychological health and well-being during a public health crisis.

## A Longitudinal Examination of the Association Between Meaning in Life, Resilience, and Mental Well-Being in Times of Coronavirus Pandemic

The Coronavirus Disease 2019 (COVID-19) was initially identified in Wuhan, China, in early December 2019 and caused over 134 million infected people and over 2,907,900 deaths worldwide as of April 10, 2021 (World Health Organization, 2020). The WHO has declared it a pandemic on March 11, 2020. As many other countries, following the pandemic, the Turkish government has taken various measures to prevent and reduce the negative impacts of the COVID-19 pandemic. The key measures taken by the government included tightening border control and implementing social distancing measures. Partial lockdown, covering the largest 30 cities alongside some smaller cities with an increasing number of cases, was announced on April 20, 2020. The lockdown required people to stay at home with restrictions of free movement (e.g., essential needs) when going out (Yıldırım and Arslan, 2020). Lockdown measures not only drastically changed people's lives but also their physical health and mental well-being.

### Mental Well-Being

Mental well-being refers to not only the absence of mental illness but also the presence of various psychosocial resources that contribute to the realization of one's full potential (Lamers et al., 2011). Mental well-being can be best described within Keyes's model, which proposes that mental well-being comprises of three dimensions: emotional, psychological, and social (2002) (Keyes, 2002). The emotional dimension reflects the frequency of positive affect and satisfaction with life (Diener et al., 1985). Psychological dimension represents one's interpersonal and intrapersonal functioning (Ryff and Keyes, 1995; Keyes et al., 2002). The social well-being dimension focuses on how well a person functions in society (Keyes, 1998). The three dimensions are distinct both theoretically and empirically yet related (Keyes et al., 2008).

Research conducted during the current pandemic has primarily focused on treatments and vaccines for COVID-19. There is some research examining the impact of the pandemic on mental health and well-being of young adults, general public, and health-care professionals in Turkey (Arslan et al., 2020a; Yıldırım and Güler, 2020; Yıldırım and Solmaz, 2020; Yıldırım et al., 2020b). The COVID-19 pandemic can cause concern about individuals' physical and mental health and well-being. The intense negative emotions that predominate during a pandemic can undoubtedly be overwhelming for people's mental health and well-being, particularly for those who have pre-existing mental health disorders, such as intense feelings of anxiety, depression, and other mental health disorders (Chatterjee et al., 2020).

### Meaning in Life and Mental Well-Being

Life is full of simultaneous experiences of pain, suffering, and sorrow, as well as joy, peace, and hope. During stressful times, people may feel overwhelmed and overstressed (Arslan et al., 2020b). Sense of meaning in life or meaning in life that people experience in the pandemic context may be damaged. Meaning in life focuses on a balanced understanding of the good life, embracing the dynamic interaction between positives and negatives, meaning-centered, and culture (Wong, 2010, 2016). According to Frankl (2006), meaning in life is an essential element of human existence, and it essentially associates with the immense existential power of a man to cope with adversities. Feeling disenchanted and disengaged, and loss of meaning in life, can be apparent during tough times and can cause hopelessness, depression, and boredom (Frankl, 2006; Wong, 2012). Frankl (2006) has highlighted that the most challenging psychological issue that individuals in the modern world experience are existential emptiness because of a lack of meaning in life, and he proposed Logotherapy to address this issue. While hard times can have adverse effects on people's psychological health and mental well-being, they can still cope with difficulties effectively by finding meaning in life.

Research conducted during the COVID-19 pandemic has also shown that meaning in life is positively related to the experience of positive affect, psychological health, and resilience and negatively associated with the experience of negative affect during the COVID-19 pandemic (Yıldırım et al., 2020a). Greater level of meaning in life was associated with lower anxiety and emotional distress during the COVID-19 health crisis (Trzebiński et al., 2020). Trzebiński et al. (2020) suggest that meaning in life may function to buffer stress reactions to the COVID-19 pandemic and that meaning in life may deteriorate in case of the long-lasting effect of the crisis. In addition, having a clear purpose or meaning in life has been indicated to have various benefits for mental and physical well-being, such as discovering values and passion, goal attainment, and focusing on the present and future social life (Schippers and Ziegler, 2019). This suggests that meaning in life can assist people to overcome the psychological effects of the pandemic crisis.

### Resilience, Meaning in Life, and Mental Well-Being

Most people may experience a loss or potentially traumatic events at some point of their lives. However, they continue to function effectively by experiencing more positive emotions and less negative emotions and disruptions in their abilities to function (Bonanno, 2004). Resilience may help people to cope with traumatic events and distress. Resilience has a complex nature identified in many ways in the literature, such as adapting successfully to adversities and growing despite adverse events (Southwick et al., 2014). Masten (2014) has emphasized a definition of resilience as “…the capacity of a dynamic system to adapt successfully to disturbances that threaten system function, viability, or development” (p. 7). Resilience also refers to the ability to resist disruption of healthy functioning in the face of adversities, by prediction and preparation (Bonanno, 2004; Chan et al., 2006). Those with a high level of resilience have various positive characteristics, including self-enhancement, optimism, hardiness, and healthy coping strategies, and report fewer mental health outcomes, such as depression, anxiety, post-traumatic stress disorders, and other sorts of psychopathology (Bonanno, 2004). Resilient individuals also report greater satisfaction with life, affect balance, and flourish (Yıldırım, 2019; Yıldırım and Belen, 2019). For example, a study with Turkish adults showed that resilient individuals had greater subjective well-being and flourished.

Studies found that resilience was a significant predictor of better general well-being and lower mental health problems (Gao et al., 2017). Resilience also mediated the relationship between psychological resources (e.g., social support) and satisfaction with life (Yıldırım and Çelik Tanrıverdi, 2020). Evidence from the pandemic context documented that resilience is a pivotal resource to promote positive psychological functioning. For example, personal resilience was found to buffer negative effects of demographic and health-related variables on mental health and stress at the beginning of the COVID-19 outbreak in Slovenia (Kavčič et al., 2020). Yıldırım and Arslan (2020) reported the mediating role of resilience in the relationship between dispositional hope and subjective well-being and psychological health among Turkish adults during the early stage of the pandemic. Furthermore, resilience acted as an effective mediator between meaning in life and psychological health as well as between the positive affect and negative affect and psychological health (Yıldırım et al., 2020a). As such, it is evident that resilience is an important psychological resource to protect the well-being and mental health of people during difficult times.

### Present Study

At the time of writing this manuscript, to the best of our knowledge, there are no published studies on the longitudinal associations between meaning in life, resilience, and mental well-being among Turkish youth. Much of the research investigating the associations between meaning in life, resilience, and mental well-being have been studied using the cross-sectional approach, with few focusing on the impact of meaning in life on mental well-being over time and the underlying mechanism between those variables. In this study, we used a longitudinal design with mediational approach to address the longitudinal relationships and underlying mechanism between meaning in life and mental well-being outcomes by focusing on resilience as a mediator. This study was set out to address the longitudinal effects of meaning in life and resilience on Turkish young adults' mental well-being. As such, this paper aims to examine how meaning in life longitudinally contributes to mental well-being amongst the Turkish youth as well as investigates the mediating role of resilience in the relationship of the meaning in life with mental well-being. To that end, we tested the following hypotheses:

(H1) to examine whether meaning in life in Wave 1 had significant effect on resilience, emotional well-being, psychological well-being, and social well-being in Wave 2, and (H2) whether resilience in Wave 2 mediated the effect of meaning in life in Wave 1 on emotional well-being, psychological well-being, and social well-being in Wave 2.

## Method

### Participants

The sample of the study comprised of 172 undergraduate students (72% women) in a public university in an urban city in Turkey. Participants ranged in age between 18 and 40 years (*M* = 20.87, *SD* = 3.92). Regarding the socioeconomic characteristics, students self-identified their socioeconomic status (SES) as follows: lower = 20.5%, middle = 49.4%, and upper = 30.1%, and no ethnic differences were reported among them. Of the initial sample, which was collected before the coronavirus pandemic (February 3–7, 2020), 385 college students participated in the first wave of the study, but 192 of the first sample completed the survey in the second wave of the study that was collected during the pandemic (June 8–12, 2020). Additionally, data from 20 young adults were removed because of either missing ID numbers at the second wave (*n* = 14) or missing or poorly completed surveys (*n* = 6). A web-based survey was created using the study measures and demographic variables. Before starting the survey, a consent form, which presented the aims of the study and informed the students, was assigned by participants.

### Measures

#### Meaningful Living Measure

The MLM is a five-item self-report scale developed to assess a sense of meaning in life among Turkish people (Arslan, 2020). All items of the scale are scored based on a five-point scale, ranging from strongly disagree to strongly agree (e.g., “I find a meaning and purpose in the difficulties that I experience”). Previous research indicated that the scale was psychometrically adequate with a strong internal reliability estimate (Arslan, 2020). The findings of this study also indicated that the scale had a strong internal reliability estimate (α = 0.88–0.90).

#### Brief Resilience Scale

The BRS was used to assess individuals' abilities to bounce back from adversity. The BRS is a six-item self-report scale that is scored using a five-point Likert scale, ranging from strongly disagree (1) to strongly agree (5; Smith et al., 2008). Previous research reported that the scale provided a strong internal reliability estimate with the Turkish sample (Dogan, 2015). Results from this study also showed that the scale had a strong internal reliability estimate (α = 0.82).

#### Mental Health Continuum (MHC-SF)

The MHC-SF is a 14-item self-report scale developed to measure people's emotional, social, and psychological well-being representing the level of mental well-being (e.g., “In the past month, how often did you feel that our society is becoming a better place for people?”; Keyes et al., 2008). All items in the scale are rated based on a six-point Likert scale, ranging between *never* (0) and *every day* (5). Previous research has shown that the scale had strong internal reliability estimates in Turkish culture (Demirci and Akin, 2015). The MHC-SF had also strong internal reliability estimates with the present sample (α range = 0.80–0.87).

### Analytic Process

Data analyses were conducted into two steps. As the first step of the analyses, preliminary analyses were carried out to explore descriptive statistics, analysis assumptions, and the association between the variables in the study. We examined the normality using kurtosis and skewness values, and their scores < |1| are acceptable for a normal distribution (Field, 2013). Pearson correlation coefficient was moreover employed to investigate the associations between the study variables. As the second step of the analyses, mediation analyses were conducted to understand the mediating effect of resilience during the coronavirus pandemic (hereinafter called Wave 2) on the relationship between meaning in life before the pandemic (hereinafter called Wave 1) and mental well-being during the pandemic. Mediation models were tested using the PROCESS macro (Model 4) version 3.5 for SPSS (Hayes, 2018). The bootstrap approach with 10,000 resamples to estimate the 95% confidence intervals was examined for the significance of indirect effect (Preacher and Hayes, 2008; Hayes, 2018). All study analyses were employed using SPSS version 25.

## Results

Descriptive statistics, internal reliability estimates, and correlation analysis results for the study variables are presented in Table 1. Preliminary analyses showed that the scores of skewness and kurtosis ranged between −1.63 and 3.86, indicating that all measures in the study had relatively normal distribution. Further analyses revealed that meaning in life at Wave 1 had significant and positive associations with resilience and psychological, emotional, and social well-being at Wave 2. Similarly, resilience at Wave 2 was also significantly correlated with psychological, emotional, and social well-being at Wave 2, as shown in Table 1.

**Table 1:** Descriptive statistics and correlations for study variables.

**Variables**	***M***	***SD***	**Skew**.	**Kurt**.	**α**	**1**.	**3**.	**4**.	**5**.	**6**.
1. ML-W1	34.96	5.41	−1.56	3.86	0.90	–				
2. RS-W2	19.10	5.62	−0.21	−0.35	0.82	0.20	–			
3. EW-W2	10.08	2.91	−0.53	0.36	0.86	0.32	0.46	–		
4. SW-W2	16.029	5.29	−0.58	−0.07	0.80	0.33	0.39	0.74	–	
5. PW-W2	22.56	5.39	−0.92	0.82	0.87	0.39	0.44	0.68	0.74	–

*All correlations are significant at the 0.001 level (2-tailed)*.

**Figure 1:**
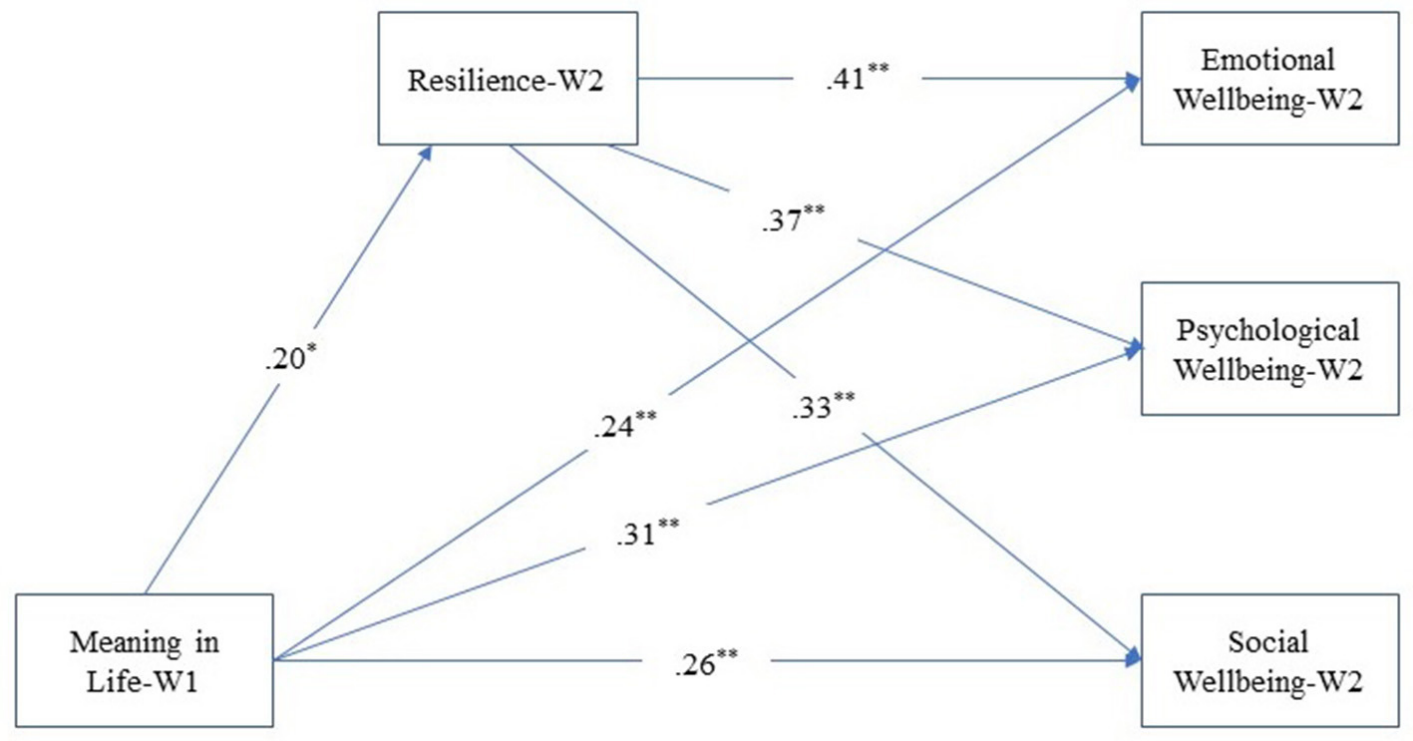
Mediation model indicating the relationships between the variables of the study. *p < 0.05; **p < 0.01.

We examined the mediating role of resilience at Wave 2 during the pandemic in the association between meaning in life at Wave 1 and psychological, emotional, and social well-being at Wave 2. Findings from the mediation model showed that meaning in life Wave 1 had significant predictive effects on resilience (*β* = 0.20, *p* < 0.05), and psychological (*β* = 0.31, *p* < 0.01), emotional (*β* = 0.24, *p* < 0.01) and social well-being (*β* = 0.26, *p* < 0.01) at Wave 2 as seen in Figure 1. Resilience partially mediated the effect of meaning in life on people's psychological (standardized indirect effect = 0.07; BootSE = 0.03, BootLLCI–ULCI = 0.02–0.14), emotional (standardized indirect effect = 0.08; BootSE = 0.03, BootLLCI–ULCI = 0.02–0.15), and social well-being (standardized indirect effect = 0.06; BootSE = 0.03, BootLLCI–ULCI = 0.01–0.13). Meaning in life explained 4% of the variance in resilience, both variables together accounted for 29% the variance in psychological well-being, 27% the variance in emotional well-being, and 22% the variance in social well-being. Unstandardized estimates for the mediation models and standardized indirect effects are presented in Table 2.

**Table 2:** Unstandardized coefficients for the mediation models.

	**Consequent**			
	***M1*** **(Resilience-W2)**			
**Antecedent**	**Coeff**.	***SE***	***t***	***p***
*X* (Meaning in life-W1)	0.20	0.07	2.61	0.009
Constant	11.97	2.76	4.33	<0.001
	*R*^2^ = 0.04			
	*F* = 6.82; *p* < 0.05			
	***Y**_**1**_* **(Emotional well-being-W2)**			
*X* (Meaning in life-W1)	0.13	0.04	3.65	0.293
*M2* (Resilience)	0.21	0.03	6.19	<0.001
Constant	1.38	1.31	1.05	<0.001
	*R*^2^ = 0.27			
	*F* = 31.54; *p* < 0.001			
	***Y**_**2**_* **(Psychological well-being-W2)**			
*X* (Meaning in life-W1)	0.31	0.07	4.73	<0.001
*M2* (Resilience)	0.36	0.06	5.65	<0.001
Constant	4.78	2.41	1.98	0.049
	*R*^2^ = 0.29			
	*F* = 33.76; *p* < 0.001			
	***Y**_**3**_* **(Social well-being-W2)**			
*X* (Meaning in life-W1)	0.26	0.07	3.81	<0.001
*M2* (Resilience)	0.31	0.06	4.80	<0.001
Constant	0.97	2.48	0.39	0.695
	*R*^2^ = 0.22			
	*F* = 23.25; *p* < 0.001			

*SE, standard error; Coeff, unstandardized coefficient; X, independent variable; M, mediator variables; Y, outcomes or dependent variables*.

## Discussion

The present paper aimed to investigate how meaning in life longitudinally contributed to mental well-being amongst the Turkish young adults as well as examined the mediating effect of resilience in the relationship of meaning in life with the components of mental well-being. We investigated the mediating role of resilience at Wave 2 during the pandemic in the association between meaning in life at Wave 1 and psychological, emotional, and social well-being at Wave 2. Results from the study showed that meaning in life in Wave 1 had significant predictive effects on resilience and mental well-being at Wave 2. Resilience partially mediated the effect of meaning in life on young people's psychological, emotional, and social well-being.

Meaning in life is a balanced understanding of the good life and dynamic interaction between positives and negatives (Wong, 2010, 2016). Considering the Logotherapy and the second wave of positive psychology approach (Frankl, 2006; Wong, 2012), this sense is an important existential source that contributes to coping with adverse circumstances, such as coronavirus pandemic experiences ( Yıldırım et al., 2020a; Arslan and Yıldırım, 2021). Specifically, feeling disenchanted and disengaged, loss of the sense of meaning in life can cause psychological challenges, such as depression, during this challenging time (Frankl, 2006; Wong, 2012). During difficult times, people might also cope with the adverse impacts of these experiences effectively by using a sense of meaningful life to protect their mental health and well-being. Additionally, the results indicated the longitudinal predictive effect of meaning in life on resilience during the pandemic. These results indicate that meaning in life helps people to deal with the stressors particularly in the context of pandemic challenges and promote their mental well-being by helping them to move beyond not only to survive but also to a new level of resilience (Arslan et al., 2020a; Yıldırım et al., 2020a). Therefore, a meaningful life might contribute to building protective factors that facilitate people's resilience, which in turn enhances their mental health and well-being. The results of the study were also consistent with previous research performed during the pandemic, showing the relationship between the meaning in life and mental well-being and resilience (Arslan et al., 2020a; Trzebiński et al., 2020; Yıldırım et al., 2020a; Arslan and Yıldırım, 2021). Previous studies were consistent with these findings, indicating that meaning in life was closely associated with mental well-being indicators (Trzebiński et al., 2020; Arslan and Allen, 2021; Yıldırım et al., 2021). In a cross-sectional study, for example, Arslan et al. (2020a) found that meaning in life was a significant predictive factor of mental well-being among Turkish young adults. Having a sense of meaning and purpose in life was also associated with various benefits for mental well-being such as discovering values and passion, goal attainment, and focusing on the present and future social life (Schippers and Ziegler, 2019). The sense of meaning in life might facilitate the ability to bounce back and overcome the adverse pandemic experiences. Hereby, the study results indicate that young adults with high levels of meaning in life have higher levels of resilience against pandemic challenges.

The results revealed that resilience mediated the predictive effect of meaning in life in Wave 1 on social, emotional, and psychological well-being in Wave 2. This finding suggests that that resilience mediates the effect of meaning in life before the pandemic on the mental well-being of young adults. Although most people are more likely to experience losses or potentially traumatic events at some point in their lives during the COVID-19 pandemic, some of them can adapt successfully and continue to function effectively by experiencing more resilience (Bonanno, 2004; Chan et al., 2006). That is, resilience may help them to deal with these adverse experiences and distress. Resilience is a powerful personal resource that helps people to cope with the pandemic challenges (Yıldırım et al., 2020a), which in turn improves their mental health and well-being. Consistent with the results of this study, past research showed that resilience was significantly associated with positive (e.g., life satisfaction, psychological well-being) and negative (e.g., depression, anxiety) mental well-being indicators (Bonanno, 2004; Arslan, 2015; Arslan and Balkis, 2016; Kansky and Diener, 2017; Cohen et al., 2020; Georgoulas-Sherry, 2020; McDonnell and Semkovska, 2020; Yıldırım and Arslan, 2020; Yıldırım and Çelik Tanrıverdi, 2020). For example, Gao et al. (2017) reported that resilience was a significant predictor of better general well-being and lower mental health problems. Resilience was also found as a mediator in the relationship between psychological resources (e.g., social support) and satisfaction with life (Yıldırım and Çelik Tanrıverdi, 2020). Similarly, studies on the pandemic indicated that resilience is an essential resource to promote positive psychological functioning (Kavčič et al., 2020; Arslan and Yıldırım, 2021). Furthermore, resilience acted as an effective mediator in the association between meaning in life and psychological health problems as well as between the positive affect and negative affect and psychological health problems (Yıldırım et al., 2020a). People with high levels of resilience might use more adaptive strategies to overcome challenges and maintain positive adaptation in the site of these experiences (Arslan, 2020). Therefore, resilience might modify the adverse effect of the coronavirus pandemic on their mental well-being.

## Limitations and Implications

The present study involves some limitations. First, this study was conducted using self-reported measures that are considered an important limitation of the study. Therefore, future research using multiple techniques (e.g., qualitative and quantitative) should be conducted to provide additional insights into the relationship between the study variables. Second, data was collected from college students, and this is considered another limitation of the study. Given the characteristics of the study sample, future studies could be performed using different and large samples to examine the associations that were reported in this study. Subsequently, further research is still needed to examine resilience from the perspective of the second wave of positive psychology, where individuals are trained to adopt the attitude of embracing suffering (Yıldırım et al., 2020a). Finally, we used unidimensional measures of meaning in life and resilience in this study. Given the multidimensional nature of meaning in life and resilience, using a multidimensional strategy toward operationalizing meaning in life and resilience could be useful to investigate the links between the variables of this study in future research.

Despite these limitations noted above, results from this study provide some implications for research and practice in the light of the second wave of positive psychology in overcoming the impacts of coronavirus challenges. Findings from the study showed that meaning in life had a longitudinal predictive effect on resilience and mental well-being indicators, and resilience mediated the effect of meaning in life before the pandemic on young adults' mental well-being during the pandemic. These results indicate that meaning in life is an essential aspect of implementing resilience-centered interventions, and mental health providers could use meaning-centered strategies not only to increase young adults' sense of meaning and purpose in life but also to build up resilience to improve their mental well-being. Mental health providers could be designed prevention and intervention services to improve the ability to thrive in the face of the negative impacts of coronavirus to promote mental well-being, as well as reduce the risk of negative indicators of mental well-being. Specifically, meaning-centered strategies could be key to improve the ability to overcome and adapt successfully to adversities. This approach may facilitate young adults to cope with challenges (e.g., coronavirus pandemic) by fostering their resilience and protective resources.

## Data Availability Statement

The raw data supporting the conclusions of this article will be made available by the authors, without undue reservation.

## Ethics Statement

The studies involving human participants were reviewed and approved by Mehmet Akif Ersoy University. The patients/participants provided their written informed consent to participate in this study.

## Author Contributions

GA and MY contributed to the design of the study. GA analyzed the data and wrote the method, results, and discussion sections. MY wrote the introduction. All authors contributed to manuscript revisions, read, and approved the final version of the manuscript, agreed to be accountable for the content of the work, and approved the submitted version of the article.

## Conflict of Interest

The authors declare that the research was conducted in the absence of any commercial or financial relationships that could be construed as a potential conflict of interest.
